# Supplementary material for: Precise phylogenetic analysis of microbial isolates and genomes from metagenomes using PhyloPhlAn 3.0
Source: Nat Commun. 2020 May 19;11:2500. doi: 10.1038/s41467-020-16366-7 (PMC7237447; doi:10.1038/s41467-020-16366-7)
Supplement: Supplementary file 1 — Supplementary Information [file 41467_2020_16366_MOESM1_ESM.pdf]

Precise phylogenetic analysis of microbial isolates and genomes from metagenomes using PhyloPhlAn 3.0

Asnicar et al.

Supplementary Information

Supplementary Figures

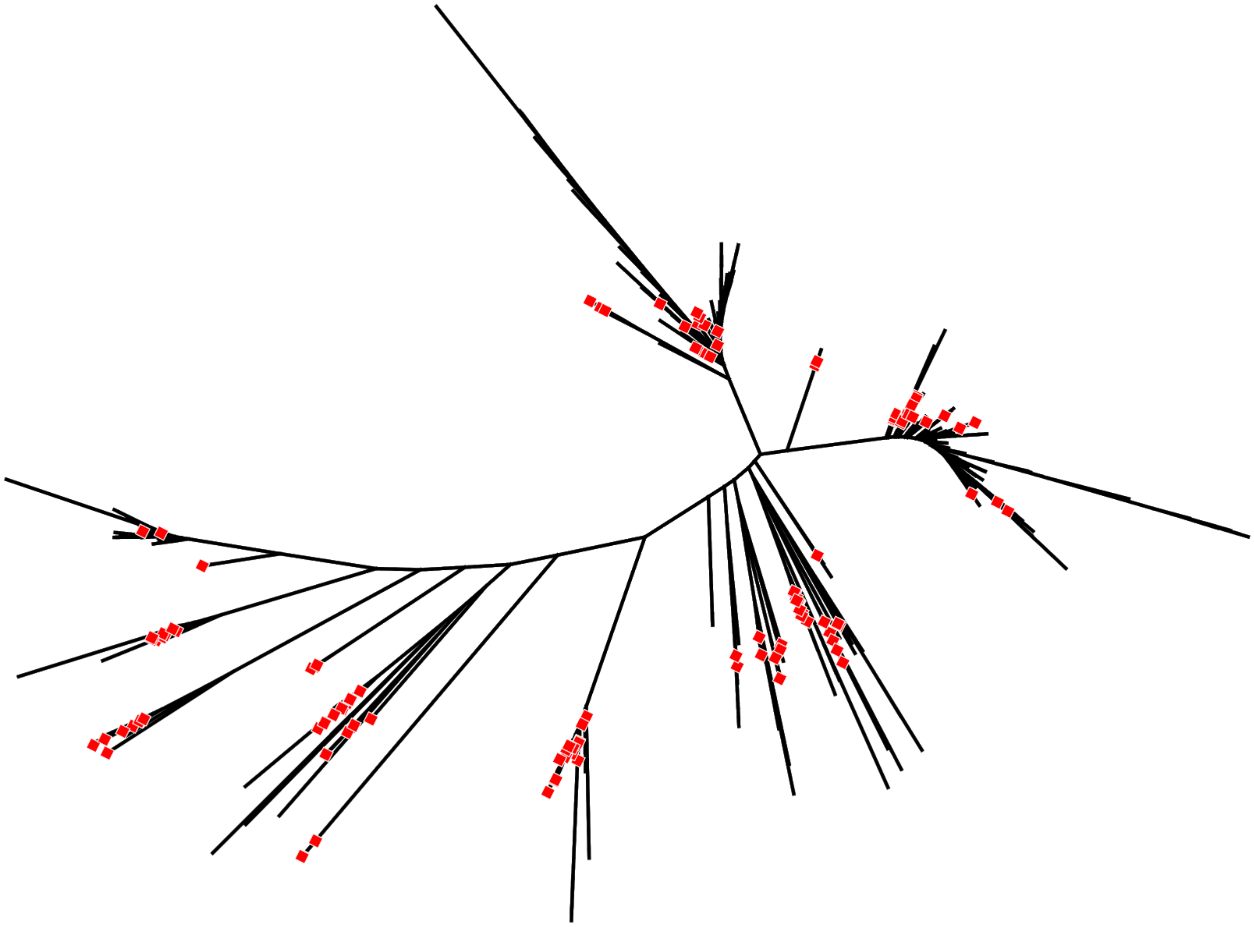

Supplementary Figure 1. Unrooted phylogeny of *Staphylococcus aureus* including the 135 isolates and a 1,000 reference genomes. Highlighted in red the 135 isolates we previously identified in [1]. The phylogeny reconstructed with PhyloPhlAn 3.0 and based on 1,658 core UniRef90 proteins, is showing how the 135 *S. aureus* isolate genomes are representing a good species diversity, being well distributed and placed in the phylogeny along with the 1,000 reference genomes.

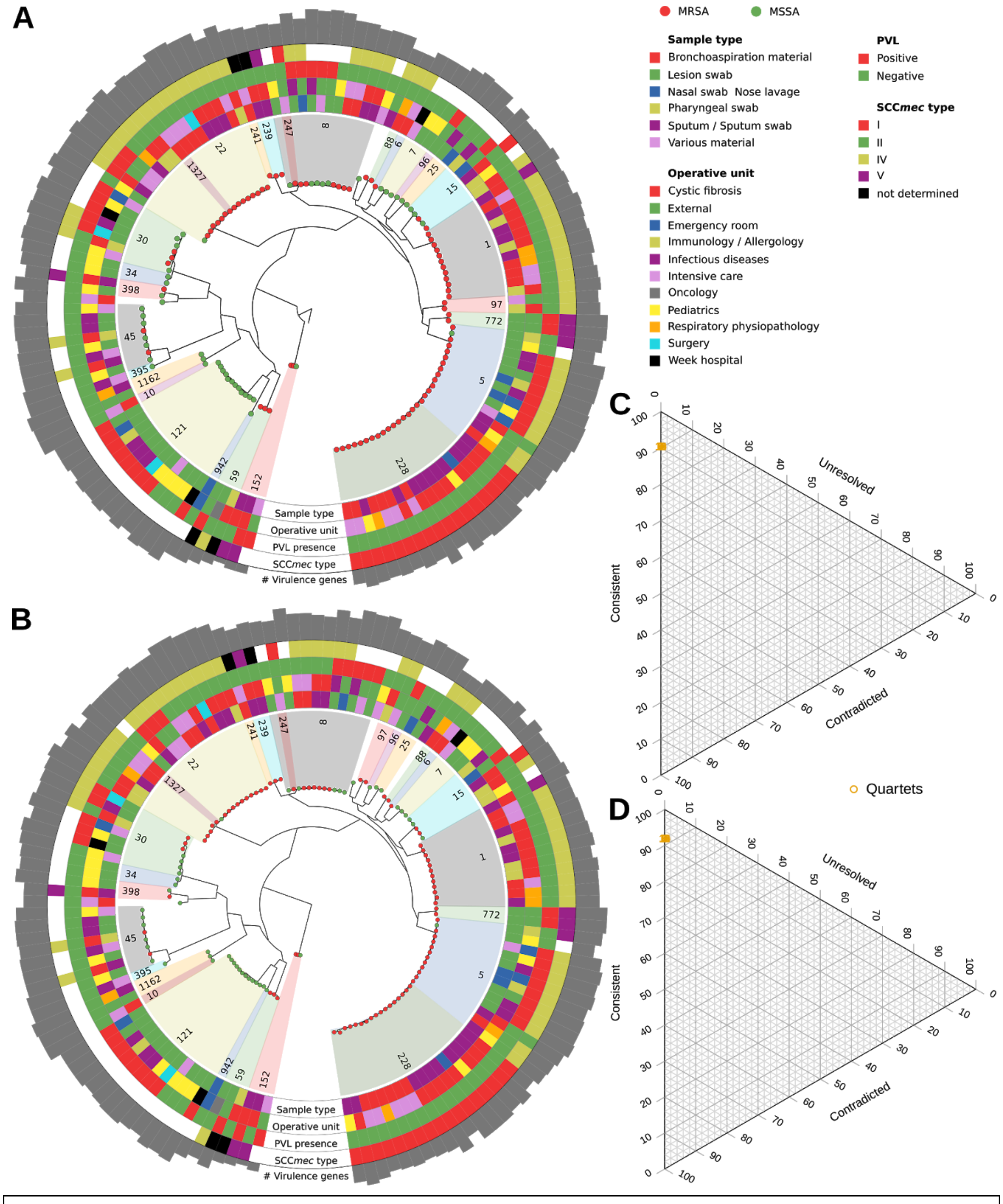

Supplementary Figure 2. Side-by-side comparison of *Staphylococcus aureus* phylogenies. (A) The original manually curated [1] phylogeny. **(B)** The phylogeny automatically reconstructed by PhyloPhlAn 3.0 and visualized with GraPhlAn using the same annotations of (A). **(C)** Ternary plot of quartet distances showing the consistency between *S. aureus* phylogenies. **(D)** Ternary plot of quartet distances of the two *S. aureus* phylogenies reduced to a single genome for each sequence type.

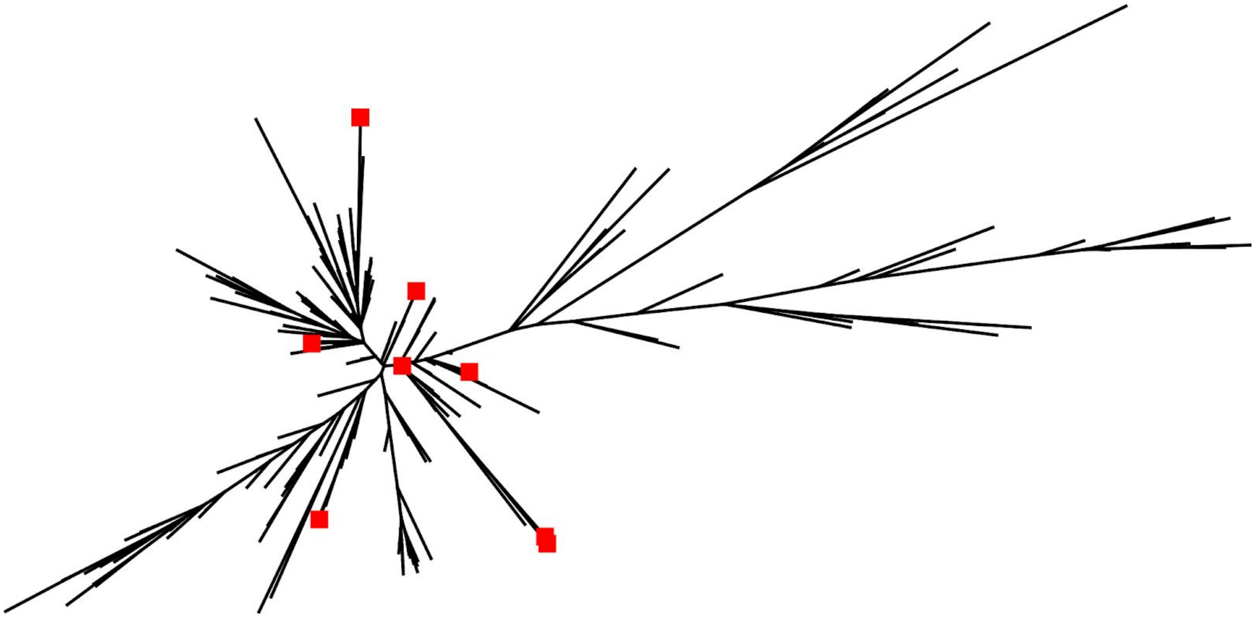

Supplementary Figure 3. Unrooted phylogeny of *Escherichia coli* that includes 200 reference genomes and the eight Ethiopian MAGs. Highlighted in red the eight *E. coli* MAGs in the phylogenetic context of 200 *E. coli* reference genomes. The phylogeny has been reconstructed with PhyloPhlAn 3.0 and it is based on 3,246 core UniRef90 proteins.

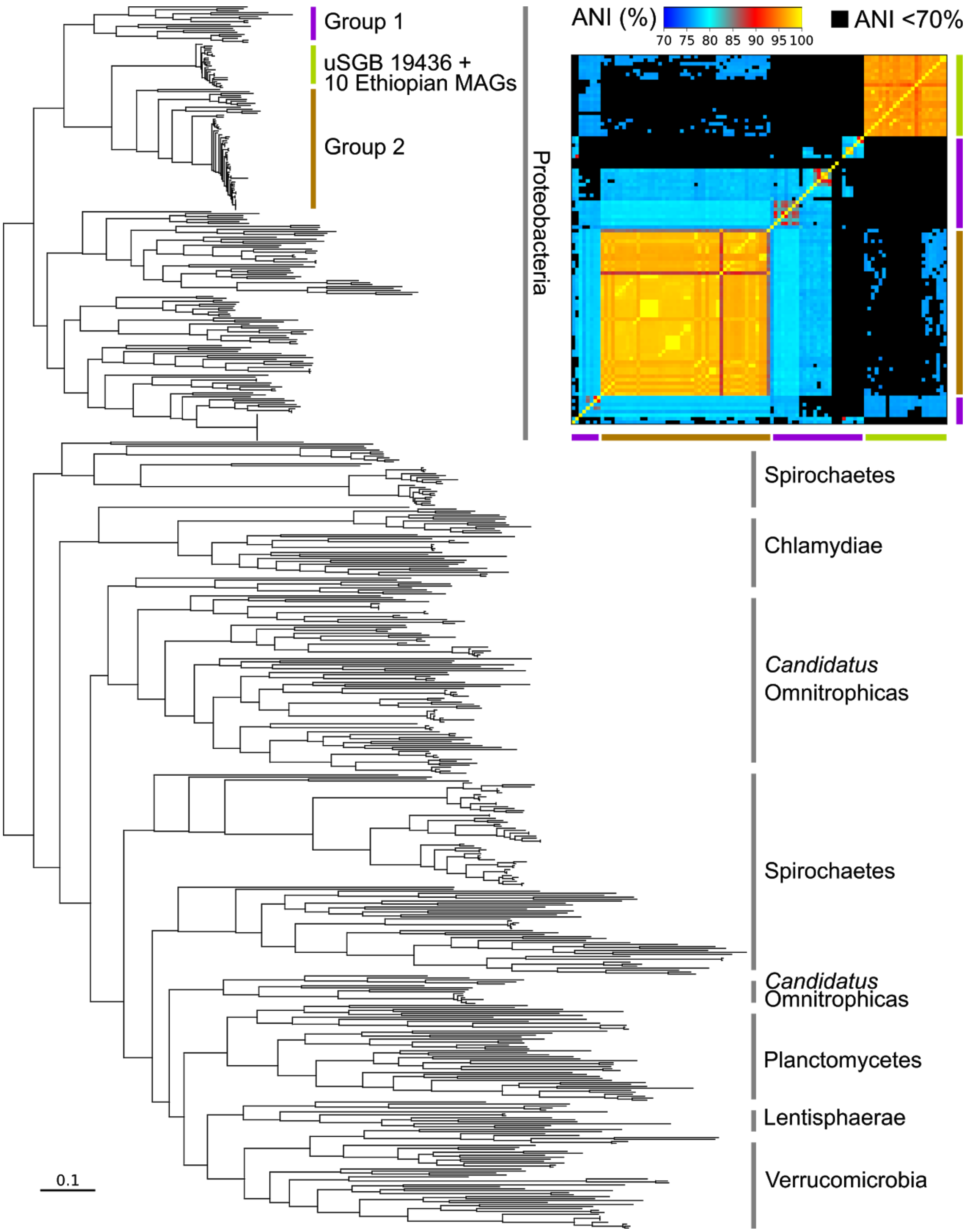

Supplementary Figure 4. Uncollapsed phylogeny of uSGB 19436 with its closest phyla. Uncollapsed phylogeny of the Ethiopian MAGs assigned to the Proteobacteria phylum (class Epsilonproteobacteria) together with the genomes reconstructed in [2] assigned to uSGB 19436 and the 589 genomes from the closest phyla: Proteobacteria (Epsilonproteobacteria, non-monophyletic with the Proteobacteria), Spirochaetes, Chlamydiae, Planctomycetes, *Candidatus* Omintrophica, Lentisphaerae, and Verrucomicrobia. In the top-right inset, genomes are compared using the average nucleotide identity (ANI) measure.

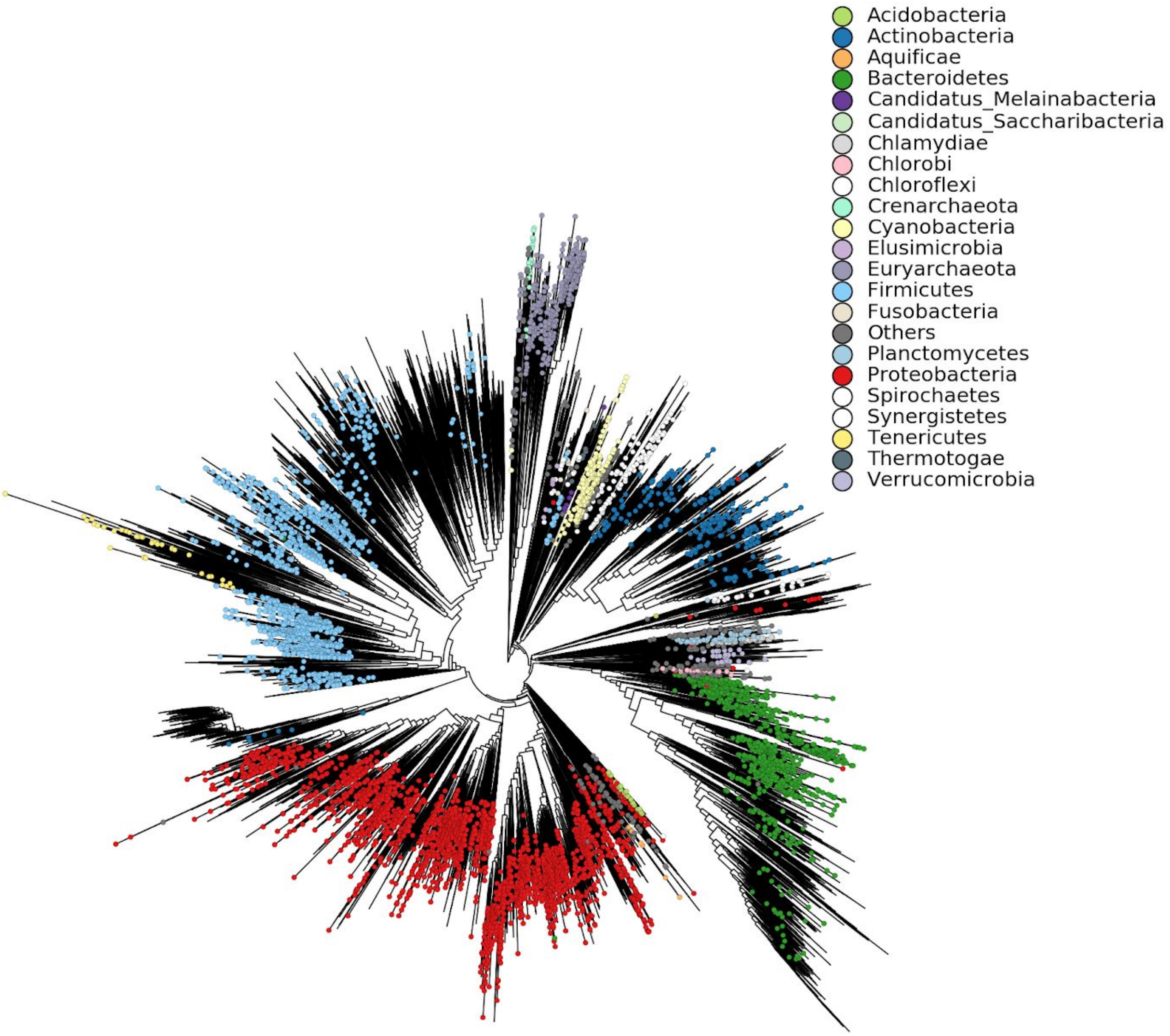

Supplementary Figure 5. PhyloPhlAn 3.0 tree-of-life highlighting non-human SGBs. The phylogeny is the same as shown in **Fig. 4**, but highlights such SGBs in which there is no present a MAG reconstructed from a human sample.

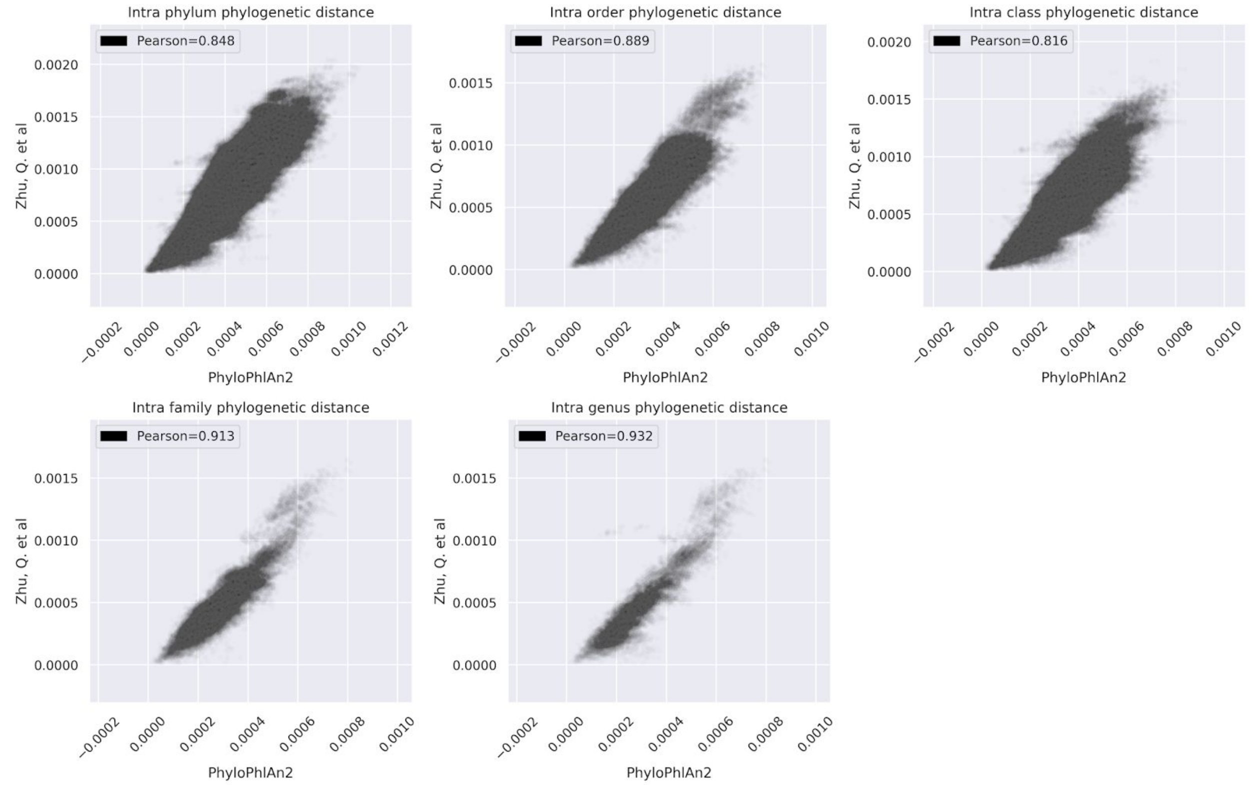

Supplementary Figure 6. Pairwise phylogenetic distances by taxonomic levels between the PhyloPhlAn 3.0 tree of life and the one proposed in [3]. The five scatterplots show the phylogenetic distances between the same genomes present in both the PhyloPhlAn 3.0 tree of life and the tree of life reconstructed using a gene tree reconciliation approach [3]. This shows a general agreement between the two trees of life, regardless of the approach employed (i.e., concatenation vs. gene tree). In each plot, the Pearson correlation coefficient is reported.

Supplementary References

1. Manara, S. et al. Whole-genome epidemiology, characterisation, and phylogenetic reconstruction of Staphylococcus aureus strains in a paediatric hospital. Genome Med. 10, 82 (2018).
2. Pasolli, E. et al. Extensive Unexplored Human Microbiome Diversity Revealed by Over 150,000 Genomes from Metagenomes Spanning Age, Geography, and Lifestyle. Cell 176, 649–662.e20 (2019).
3. Zhu, Q. et al. Phylogenomics of 10,575 genomes reveals evolutionary proximity between domains Bacteria and Archaea. Nat. Commun. 10, 5477 (2019).
4. Segata, N., Börnigen, D., Morgan, X. C. & Huttenhower, C. PhyloPhlAn is a new method for improved phylogenetic and taxonomic placement of microbes. Nat. Commun. 4, 2304 (2013).
5. Zou, Y. et al. 1,520 reference genomes from cultivated human gut bacteria enable functional microbiome analyses. Nat. Biotechnol. 37, 179–185 (2019).
