# Supplementary material for: Precise phylogenetic analysis of microbial isolates and genomes from metagenomes using PhyloPhlAn 3.0
Source: Nat Commun. 2020 May 19;11:2500. doi: 10.1038/s41467-020-16366-7 (PMC7237447; doi:10.1038/s41467-020-16366-7)
Supplement: Supplementary file 3 — Description of Additional Supplementary Files [file 41467_2020_16366_MOESM3_ESM.pdf]

Description of Additional Supplementary Files

File Name: Supplementary Data 1
Description: Comparison of the features available in PhyloPhlAn 1.0[4] and the new implemented framework PhyloPhlAn 3.0).

File Name: Supplementary Data 2
Description: Table of the taxonomic assignment inconsistencies in between EzBioclud used in [5] and PhyloPhlAn 3.0 based on the SGB resource [2].
