# Supplementary material for: Precise phylogenetic analysis of microbial isolates and genomes from metagenomes using PhyloPhlAn 3.0
Source: Nat Commun. 2020 May 19;11:2500. doi: 10.1038/s41467-020-16366-7 (PMC7237447; doi:10.1038/s41467-020-16366-7)
Supplement: Supplementary file 6 — Reporting Summary [file 41467_2020_16366_MOESM6_ESM.pdf]

nature research

Corresponding author(s): Nicola Segata

Last updated by author(s): Apr 17, 2020

Reporting Summary

Nature Research wishes to improve the reproducibility of the work that we publish. This form provides structure for consistency and transparency in reporting. For further information on Nature Research policies, see Authors & Referees and the Editorial Policy Checklist .

Statistics

For all statistical analyses, confirm that the following items are present in the figure legend, table legend, main text, or Methods section.

n/a	Confirmed	
x		The exact sample size (n) for each experimental group/condition, given as a discrete number and unit of measurement
x		A statement on whether measurements were taken from distinct samples or whether the same sample was measured repeatedly
	x	The statistical test(s) used AND whether they are one- or two-sided<br>*Only common tests should be described solely by name; describe more complex techniques in the Methods section.*
x		A description of all covariates tested
x		A description of any assumptions or corrections, such as tests of normality and adjustment for multiple comparisons
x		A full description of the statistical parameters including central tendency (e.g. means) or other basic estimates (e.g. regression coefficient) AND variation (e.g. standard deviation) or associated estimates of uncertainty (e.g. confidence intervals)
	x	For null hypothesis testing, the test statistic (e.g. F, t, r) with confidence intervals, effect sizes, degrees of freedom and P value noted<br>*Give P values as exact values whenever suitable.*
x		For Bayesian analysis, information on the choice of priors and Markov chain Monte Carlo settings
x		For hierarchical and complex designs, identification of the appropriate level for tests and full reporting of outcomes
	x	Estimates of effect sizes (e.g. Cohen's d, Pearson's r), indicating how they were calculated

Our web collection on statistics for biologists contains articles on many of the points above.

Software and code

Policy information about availability of computer code

Data collection	Raw metagenomes for the Ethiopian cohort are available under NCBI-SRA BioProject id PRJNA504891 and the 369 MAGs can be downloaded from the software page at http://segatalab.cibio.unitn.it/tools/phylophlan3.
Data analysis	PhyloPhlAn 3.0 is released open-source and available in GitHub at https://github.com/biobakery/phylophlan and the version used in this work is archived with DOI: http://doi.org/10.5281/zenodo.3727181.<br>diamond (version v0.9.9.110) with parameters: “blastx --quiet --threads 1 --outfmt 6 --more-sensitive --id 50 --max-hsps 35 -k 0” and with parameters: “blastp --quiet --threads 1 --outfmt 6 --more-sensitive --id 50 --max-hsps 35 -k 0”.<br>mafft (version v7.310) with the “--anysymbol” option.<br>trimal (version 1.2rev59) with the “-gappyout” option.<br>FastTree (version 2.1.9) with “-mlacc 2 -slownni -spr 4 -fastest -mlnni 4 -no2nd -gtr -nt” options.<br>RAxML (version 8.1.15) with parameters: “-m PROTCATLG -p 1989”.<br>R package "quartet" (version 1.1.0).<br>GraPhlAn (version 1.1.3)

For manuscripts utilizing custom algorithms or software that are central to the research but not yet described in published literature, software must be made available to editors/reviewers. We strongly encourage code deposition in a community repository (e.g. GitHub). See the Nature Research guidelines for submitting code & software for further information.

Data

Policy information about availability of data

All manuscripts must include a data availability statement. This statement should provide the following information, where applicable:
- Accession codes, unique identifiers, or web links for publicly available datasets
- A list of figures that have associated raw data
- A description of any restrictions on data availability

Raw metagenomes for the Ethiopian cohort are available under NCBI-SRA BioProject id PRJNA504891 [https://www.ncbi.nlm.nih.gov/bioproject/PRJNA504891] and the 369 MAGs can be downloaded from the software page at http://segatalab.cibio.unitn.it/tools/phylophlan3.

Field-specific reporting

Please select the one below that is the best fit for your research. If you are not sure, read the appropriate sections before making your selection.

☐ Life sciences ☐ Behavioural & social sciences ☒ Ecological, evolutionary & environmental sciences

For a reference copy of the document with all sections, see nature.com/documents/nr-reporting-summary-flat.pdf

Ecological, evolutionary & environmental sciences study design

All studies must disclose on these points even when the disclosure is negative.

Study description	Phylogenomic analyses of publicly available bacterial and archaeal genomes, and newly sequenced metagenomes from an Ethiopian cohort.
Research sample	Samples considered in this paper range from (1) the phylogenetic characterization of isolate genomes of Staphylococcus aureus; (2) the reconstruction of the prokaryotes tree-of-life phylogeny; (3) Phylogenetic analysis of the metagenomes of the Ethiopian cohort; (4) High-resolution phylogeny of genomes and MAGs of the Escherichia coli species; and (5) Phylogenetic characterization of an unknown species-level genome bin (SGB) assigned to the Proteobacteria phylum.<br>Genomes used in the examples above were chosen based on their taxonomic assignment, or the taxonomic label assigned by the developed method "phylophlan_metagenomic.py".<br>Genomes used in the examples above were retireved from GenBank from NCBI and through metagenomic assembly of the 50 Ethiopian metagenomes deposited under NCBI BioProject PRJNA504891.
Sampling strategy	N/A<br>Genomes were retrieved from GenBank from NCBI and through metagenomic assembly of the 50 Ethiopian metagenomes deposited under NCBI BioProject PRJNA504891. See "Research sample" section above for further specifications.
Data collection	Genomes used in the examples above were retireved from GenBank from NCBI and through metagenomic assembly of the 50 Ethiopian metagenomes deposited under NCBI BioProject PRJNA504891.
Timing and spatial scale	N/A<br>Genomes were retrieved from GenBank from NCBI and through metagenomic assembly of the 50 Ethiopian metagenomes deposited under NCBI BioProject PRJNA504891. See "Research sample" section above for further specifications.
Data exclusions	In each of the phylogenetic analysis, input genomes and MAGs were quality controlled by the internal PhyloPhlAn 3.0 preset parameters, in each of the five examples described in the "Research sample" section above with different criteria, as described in their respective tutorials available here: https://github.com/biobakery/phylophlan/wiki.<br>Pre-selection of genomes were made for the prokaryotes tree-of-life phylogeny in Figure 4, where we dereplicated prokaryotic genomes by clustering them at 5% ANI similarity, picking for each of the 19,607 clusters, only one representative genome, as also detailed in the manuscript.
Reproducibility	All the analyses in the paper are described and can be reproduced using their respective tutorials available here: https://github.com/biobakery/phylophlan/wiki.<br>For the phylogenetic trees in Figures 2A and 3C, and Supplementary Figures 1, 2B, 3, 4, the phylogeny reconstruction step was repeated 100 times for computing branches bootstrap support values. Bootstrap support values indicate how many times a branch was placed in the same position and are reported in their respective figures.
Randomization	N/A<br>We are not analyzing case/control samples, so randomization is not needed.
Blinding	N/A<br>We are not analyzing case/control samples, so blinding is not needed.

Did the study involve field work? ☐ Yes ☒ No

Reporting for specific materials, systems and methods

We require information from authors about some types of materials, experimental systems and methods used in many studies. Here, indicate whether each material, system or method listed is relevant to your study. If you are not sure if a list item applies to your research, read the appropriate section before selecting a response.

Materials & experimental systems

n/a	Involved in the study
☒	☐ Antibodies
☒	☐ Eukaryotic cell lines
☒	☐ Palaeontology
☒	☐ Animals and other organisms
☒	☐ Human research participants
☒	☐ Clinical data

Methods

n/a	Involved in the study
☒	☐ ChIP-seq
☒	☐ Flow cytometry
☒	☐ MRI-based neuroimaging
